# Supplementary material for: Asymmetric Synthesis of Quaternary Hydantoins via a Palladium-Catalyzed Aza-Heck Cyclization
Source: J Am Chem Soc. 2025 Nov 14;147(49):44692–8. doi: 10.1021/jacs.5c16022 (PMC12703750; doi:10.1021/jacs.5c16022)
Supplement: Supplementary file 2 [file ja5c16022_si_002.zip › All NMR FID Files/1-S-trans/1-S-trans_CarbonNMR/3/pdata/1/email_HO01-166A-13C_3_1.pdf]

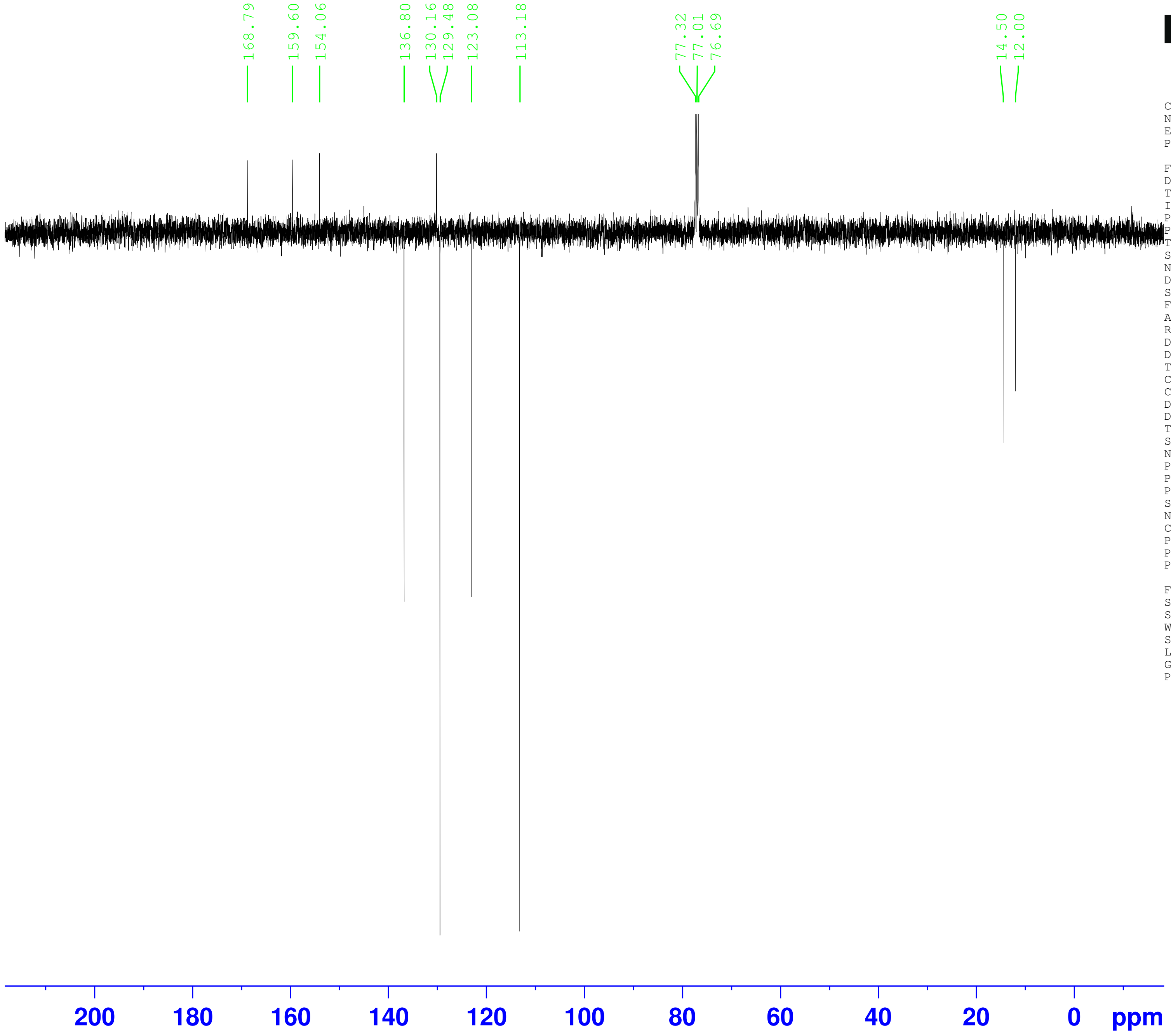

Current Data Parameters
NAME HO01-166A-13C
EXPNO 3
PROCNO 1

F2 - Acquisition Parameters
Date_ 20250628
Time 19.42 h
INSTRUM Avance Neo 400 U of DE Brown Lab
PROBHD Z104450_0412 (
PULPROG jmod
TD 65536
SOLVENT CDCl3
NS 1024
DS 4
SWH 23809.524 Hz
FIDRES 0.726609 Hz
AQ 1.3762560 sec
RG 101
DW 21.000 usec
DE 6.50 usec
TE 300.0 K
CNST2 145.0000000
CNST11 1.0000000
D1 5.00000000 sec
D20 0.00689655 sec
TD0 1
SFO1 100.6228298 MHz
NUC1 13C
P1 10.00 usec
P2 20.00 usec
PLW1 44.93500137 W
SFO2 400.1316005 MHz
NUC2 1H
CPDPRG[2 waltz65
PCPD2 90.00 usec
PLW2 8.31639957 W
PLW12 0.23101000 W

F2 - Processing parameters
SI 32768
SF 100.6127685 MHz
WDW EM
SSB 0
LB 1.00 Hz
GB 0
PC 1.40
